# Kidney Injury Molecule 1 (KIM-1) as an Early Predictor for Acute Kidney Injury in Post-Cardiopulmonary Bypass (CPB) in Open Heart Surgery Patients

**DOI:** 10.1155/2019/6265307

**Published:** 2019-03-12

**Authors:** Nora A. Khreba, Mostafa Abdelsalam, A. M. Wahab, Mohammed Sanad, Rania Elhelaly, Mohammed Adel, Ghada El-Kannishy

**Affiliations:** ^1^Mansoura Nephrology and Dialysis Unit, Internal Medicine Department, Mansoura University, Egypt; ^2^Cardiothoracic Surgery Department, Mansoura University, Egypt; ^3^Clinical Pathology Department, Mansoura University, Egypt; ^4^Anesthesia and Surgical Intensive Care, Mansoura University, Egypt

## Abstract

**Introduction:**

Postoperative acute kidney injury is associated with a higher mortality, a more complicated hospital course with longer hospital stay. Urinary kidney injury molecule 1 may play an important role as an early predictor of acute kidney injury post-cardiopulmonary in open heart surgery.

**Methods:**

We evaluated 45 patients who underwent open heart surgery from January 2016 to June 2016. Both urinary kidney injury molecule 1 and serum creatinine were evaluated before operation and 3hs and 24hs after operation. Acute kidney injury was diagnosed according to Kidney Disease: Improving Global Outcomes, 2012 guidelines.

**Results:**

In this study, 27 patients developed acute kidney injury. The three hour-post-surgery urinary kidney injury molecule 1 was significantly higher in the acute kidney injury group (P<0.015) and, at the same time, we did not find any significant difference in the serum creatinine levels between the two groups.

**Conclusion:**

Although serum creatinine is still the gold standard for diagnosis of acute kidney injury searching for other new markers is mandatory. Urinary kidney injury molecule 1 can be used as simple noninvasive and specific biomarker for early diagnosis of acute kidney injury.

## 1. Introduction

Postoperative acute kidney injury {AKI} is associated with a higher mortality, a more complicated hospital course with longer hospital stay, and a higher risk for hospital acquired infections [1]. Even slight increase in serum creatinine levels which happen in the postoperative setting has a significant reduction of survival rate and poor outcomes [2].

The risk of postsurgery AKI can be decreased by using less invasive approaches, such as off-pump coronary artery bypass grafting or transcatheter aortic valve implantation, but these options cannot be employed in all cases, as cardiac surgery remains the first and only option in many conditions such as severe coronary artery disease, valvular heart diseases, and complex interventions. Therefore, it is important to adopt strategies which help the clinician to prevent AKI or diagnose it early. Old age, preprocedural chronic kidney disease, obesity, some comorbidities, wide pulse pressure, and some pharmacological regimens represent risk factors for post-cardiopulmonary bypass {CPB} AKI, poor outcomes, and mortality [3–5].

Acute kidney injury in patients undergoing cardiac surgery ranged from 7.7% to 28.1% in different studies, according to the criteria adopted to define AKI [6]. Renal replacement therapy {RRT} is indicated in nearly 1% of patients who developed AKI [7].

Important intraoperative factor such as use and duration of CPB is closely related to postoperative AKI [8]. Postoperative efforts aimed toward maximizing cardiac output, avoiding drugs that lead to vasoconstriction of the renal artery, providing adequate crystalloid infusion, and alkalinizing urine [9].

The half-life of creatinine limits its use as a marker of renal function. As if the glomerular filtration rate {GFR} decreases, its half-life increases from 4 h to 24–72 h. Consequently, the serum concentration may increase after 24–36 h of a definite kidney injury. Moreover, in patients with sepsis, liver dysfunction, and muscle wasting, a true decrease in GFR may not be adequately assessed by serum creatinine [10].

Kidney injury molecule 1 {KIM-1} is a promising marker for early detection of AKI, and its concentration is markedly increased within hours following kidney injury [11]. It is a type I transmembrane glycoprotein with two extracellular domains. After injury, the extracellular domains of KIM-1 separate from the cell surface and enter the urine [12]. KIM-1 expression is low in normal kidneys but is significantly increased in proximal tubule cells following AKI.

In our study, we aimed to assess the role of urinary KIM-1 level for early detection of AKI in postoperative cardiac patients.

## 2. Materials & Methods

### 2.1. Ethical Issues

The current study was approved by the Institutional Research Board of Mansoura Faculty of Medicine. Free voluntary informed consent was taken from all patients. Confidentiality and privacy were considered as regards personal, clinical, and laboratory data.

### 2.2. Patients Selection

In this prospective cohort study, 45 patients {23  males  and  22  females} who admitted to cardiothoracic surgery department in Mansoura University Hospital in Egypt and underwent cardiopulmonary bypass were enrolled in our study from January 2016 to June 2016.

Patients with normal kidneys seen by pelvi-abdominal ultrasound and normal kidney functions approved by laboratory investigations and with estimated glomerular filtration rates higher than 90 ml/min by the Modification of Diet in Renal Disease MDRD equation were included.

### 2.3. Study Design

From all participants before operation and 3h and 24h after operation, 5ml of venous blood samples was withdrawn from each patient. Blood samples were allowed to clot for 15 minutes and then serum was separated by centrifugation for 10 minutes at 6000 rpm. Serum creatinine, sodium, and potassium were assessed immediately with daily follow-up of serum creatinine till the time of discharge on Dimension Xpand plus {Seimens Healthcare GmbH, Henkestr. 127- 91052 Erlangen, Germany} using its commercial kits.

For urinary KIM-1, 10 ml of midflow urinary samples was collected in disposable urine cups without preservatives at the same time points of blood sampling, before operation and 3 hours after operation. Urine samples were centrifuged at 2000-3000 rpm for 20 min and then supernatant was kept in -20°C until KIM-1 will be analyzed.

Quantitative determination of urinary KIM-1 was done using enzyme linked immunoassay {ELISA} kit supplied by Sun Red {Shangahi, China}.

### 2.4. Characteristics of Operation

Duration of CPB, cross-clamping time and core temp were recorded for all the included patients.

### 2.5. Diagnosis of AKI

Use KDIGO criteria for AKI diagnosing {2012} which include

- Increase in Serum creatinine {SCr} more than 0.3 mg/dl within 48 hours.
- Increase in SCr to more than 1.5 times of baseline SCr, which is known or presumed to have occurred within the previous 7 days.
- Urine volume less than 0.5 ml/kg/h for six hours.

Patients were divided into 2 groups: {1} AKI group included {27  patients}  {16  males  and  11  females} with cardiac disease that developed AKI after open heart surgery with cardiopulmonary bypass. {2} Non-AKI group included 18 individuals {7  males  and  11  females}.

### 2.6. Statistical Analysis

Data were verified. All statistical procedures were performed Using the Statistical Package of Social Sciences {SPSS} version 22 for Windows {SPSS, Inc., Chicago, IL, USA}. Using Kolmogorov-Smirnov test for normality, the distribution of tested variables was examined. Independent samples t- test was used to determine the significance of the differences between continuous variables with normal distribution. Mann-Whitney test was used to determine the significance of the differences between continuous variables which was not normally distributed. For comparison between qualitative variables, Chi- square or Fisher exact test was used. The mean differences between more than two groups normally distributed were analyzed by one-way analysis of variance {ANOVA}, while Kruskal Wallis test was used for nonparametric variables which were not normally distributed.

ROC curve statistics were used to estimate specificity and sensitivity of the markers. Statistic results were considered statistically significant when p values were less than 0.05.

## 3. Results

Forty-five patients were included in this study. Diabetic patients represented 33.3% while hypertensive patients were 44.4%. Open heart surgery was indicated due to ischemic heart disease, valvular heart disease, and congenital heart diseases {Table 1 }.

In this study, 27 patients developed AKI according to KDIGO criteria, 2012, after 24 hours of cardiopulmonary bypass in open heart surgery.

The mean cardiopulmonary bypass duration was 132.8 minutes ± 29.03, while the median cross-clamping time was 80 minutes with range {60-170}. On the other hand, the median core temperature of patients during operation was 33°C with range {32-35}°C {Table 2 }.

Comparison between AKI and non-AKI patients revealed no statistically significant finding as regards preoperative demographic, medical, and laboratory data {Table 1 }.

In this study, we did not find any significant difference between the AKI and the non-AKI patients as regards the indications and the characteristics of the open heart surgery {Table 2 }.

There was no significant difference between the two groups as regards preoperative serum creatinine and urinary KIM-1 levels while the non-AKI group showed significantly lower urine output in comparison to AKI group. The three hour-post-surgery urinary KIM-1 was significantly higher in the AKI group and, at the same time, we did not find any significant difference in the serum creatinine levels between the two groups. On the other hand, after 24h of surgery all the patients who developed AKI showed rising of serum creatinine in concordance with KDIGO criteria, 2012, for diagnosis of AKI and the 24h-post-surgical serum creatinine was significantly higher in the AKI group in comparison to the non-AKI group. Both groups showed significant increase in the postoperative urine output with no significant difference between them {Table 3 }.

ROC analysis of urinary KIM-1 for early detection of postoperative AKI {3 h  postoperative} was done. We found that, at cutoff value of 1.9 ng/mg of urinary KIM-1, its sensitivity was 48% and specificity was 94% with AUC 0.715, Table 4.

## 4. Discussion

AKI is a frequent complication with cardiac surgery in both children and adult and it is related to poor outcomes and mortality after the operation [13]. In the current study, KIM-1 a well-known urinary biomarker of kidney injury was evaluated as an early predictor for acute kidney injury in post-cardiopulmonary bypass {CPB} in open heart surgery patients in comparison to the routinely used serum creatinine.

The unique characteristics of cardiac surgery as an AKI risk factor include CPB, aorta cross-clamping, and high doses of exogenous vasopressors. These factors alter renal perfusion, induce cycles of ischemia and reperfusion, increase oxidative damage, and increase renal and systemic inflammation resulting in higher AKI incidence after CPB [14]. In the current study, patients were classified into two groups AKI {60%}and Non-AKI {40%} according to KDIGO, 2012, definition of AKI. We did not rely on urine output in classification of the 2 groups because most of patients were polyuric after operation due to plenty of fluid infusion during and after operation.

Han et al., 2009 [15], studied AKI after CPB and found that patients with AKI were more likely to be older and AKI was also more prevalent in hypertensive and diabetic patients. Similarly, in our study the mean age of patients who progressed to AKI was 47.40±15.24 years slightly higher than nonprogressors to AKI but this difference was not statistically significant. Percentage of DM in AKI group was {33.3%} while hypertensive patients constituted 48.14% of AKI group.

Incidence of AKI varied depending on type of the operation with higher percentage in patients who underwent valvular replacement operation {64.7%} compared to {52%} in patients who underwent CABG but the difference was not statistically significant. Risk factors associated with the development of AKI after CPB include type of surgery, with valvular procedures associated with a higher risk [1]. Reference [16] found that patients undergoing coronary artery bypass grafting {CABG} with valve surgery are at higher risk of developing AKI than those undergoing simply CABG or simply valve surgery. Patients who underwent CABG with valve surgery in our study were three cases with one case progressing to AKI.

According to our results all patients had a statistically significant higher urine output 12 h after operation than before operation {p  0.001}. However, preoperative urine output was significantly higher in the AKI, while postoperative urine output showed no significance between AKI and non-AKI groups.

Postoperative polyuria can be explained by plenty of fluid infusion during and after operation. Reference [17] suggested that the paradoxical increase of postoperative urine output may be due to impairment of tubular reabsorption and heterogeneity of nephron function triggered by inflammatory and thrombotic response during CPB which should not be interpreted as a favorable sign.

The median core temperature of patients who developed AKI was lower than that in non-AKI group with no statistical significance. Reference [18] reported that patients undergoing routine CABG were cooled to 32°C and actively rewarmed to 34°C or 37°C. Those who were rewarmed to 37°C had higher postoperative levels of serum creatinine and increased incidence of renal injury; this is suggestive that active rewarming, rather than cooling is the process responsible for renal injury.

On the other hand, the mean CPB time of patients who developed AKI was higher compared to those who did not develop AKI but with no statistical significance. Similar results were concluded by [19] that found that neither duration of surgery nor duration of CPB was associated with functional cardiac surgery associated-AKI, while [20] that studied 289 patients with cardiac surgery associated AKI found that CPB time was longer in those developed AKI. This conflict in the previous studies may be related to surgery related techniques which include strict optimization of blood flow and perfusion pressure during CPB according to physiological goals to maintain adequate tissue perfusion and oxygen saturation [21]. On the other hand, a safe time cutoff has not been determined [22].

None of the AKI patients needed renal replacement therapy within the 2 weeks of follow-up. This finding interestingly agreed with that of [23] which studied 122 patients for KIM-1 as early biomarker for the diagnosis of AKI following cardiopulmonary bypass surgery and none of them needed renal replacement therapy {RRT} but we think the implementation of new strategies for long-term follow-up of this group of patients may lead to understanding the link between AKI and other risk factors such as proteinuria and end stage renal disease [24] which may lead in the future to a new reclassification of end stage renal disease [25]

In the current study there was a statistically significant increase in the median level of 24 h after operation serum creatinine in AKI group. None of AKI patients showed an absolute increase in creatinine more than 0.3 mg/dl from baseline in the first 3 h after operation. These results were in concordance with Han et al., 2009 [15], and Mishra et al., 2005 [22], who concluded that changes in serum creatinine occur late in the development of AKI typically 24 up to 48 hours after initiating surgery in the case of cardiac surgery associated AKI. At the same time, the 3h-postoperative urinary KIM-1 level was significantly high in the AKI group in comparison to non-AKI group. Surprisingly, our result was in agreement with Chertow et al., 2001 [23], Liang et al., 2010 [16], and Liangos et al., 2009 [26], who showed more than 90% sensitivity in diagnosis of AKI when KIM-1 was tested 2 h and 6 h after CPB. However, the sensitivity was decreased to 74% 12 h after CPB [27].

Results of the ROC analysis of the 3h-post-operative urinary KIM-1 level showed an AUC of 0.715. Multiple studies estimated the AUC for 3h-post-operative KIM-1 [28–30] and the AUC was 0.715, 0.71, 0.78, 0.68, and 0.6, respectively. These differences seemed to be most likely related to the patient heterogeneity, small study populations, and timing of postoperative urinary KIM-1 sampling. Also, the urinary concentration of KIM-1 is affected by multiple factors such as sepsis and use of contrast media. Moreover, the urinary concentration may be affected by common diseases such as atherosclerosis, hypertension, and diabetes mellitus [31].

In the current study, it was found that at cutoff value 1.9 ng/mg, 3h-post-operative urinary KIM-1 had sensitivity {48%} and specificity {94%}.

The current study is important for early detection of AKI in post-cardiopulmonary bypass {CPB} in Open Heart Surgery Egyptian Patients. However, it has its own limitations as other larger multicenter studies are recommended for more robustness of data and will check the data in different cardiothoracic surgery departments [32].

## 5. In Conclusion

Early diagnosis of AKI is still challenging and despite that serum creatinine is still the gold standard for diagnosis of AKI but searching for other new markers is mandatory. Urinary KIM-1 can be used as simple noninvasive and specific biomarker for early diagnosis of AKI.

## Figures and Tables

**Table 1:** Preoperative demographic, laboratory data, and indications of surgery of studied patients (n = 45).

Parameters	Group 1	Group 2	*P* value	Total
	(AKI Group)	(non-AKI)		n= 45
	n= 27	n= 18		
Age in years (Mean±SD)	47.40±15.24	44.55±13.95	0.529*∗*	46.26±14.64

Sex:				
Male, n (%)	16 (59.25%)	7(38.9%)	0.231*∗∗∗*	23 (51.1%)
Female, n (%)	11(40.75%)	11(61.1%)		22 (48.9%)

DM, n (%)	9(33.3%)	6(33.3%)	1*∗∗∗*	15 (33.3%)

Duration of DM in years (Mean±SD)	7.66±3.64	8.83±2.48	0.507*∗*	8.13±3.18

HTN, n (%)	13(48.14%)	7(38.8%)	0.760*∗∗∗*	20 (44.4%)

Duration of HTN in years (Mean±SD)	5.73±4.15	5.40±4.16	0.867*∗*	5.61±4.05

Virology:				
HCV n (%)	23(85.2%)	12(66.7%)	0.149*∗∗∗*	9 (20%)
HBV n (%)	3(11.1%)	6(33.3%)		1 (2.2%)
No HBV& HCV n (%)	1(3.7%)	0		35 (77.8%)

WBCs (10^6^/L) (Mean±SD)	7.61±2.47	7.52±2.34	0.903*∗*	7.58±2.39

Hb (gm/dL) (Mean±SD)	12.39±1.32	11.99±1.58	0.366*∗*	12.23±1.42

PLT (10^6^/L) (Mean±SD)	286.51±85.98	298.44±97.88	0.668*∗*	291.28±90.03

HCO3 (mEq/L) (Mean±SD)	22.79±2.07	22.51±2.10	0.662*∗*	22.68±2.06

Na (mEq/L) (Mean±SD)	140.03±5.17	141.66±4.56	0.285*∗*	140.68±4.95

K (mEq/L) (Mean±SD)	4.21±0.54	4.16±0.67	0.764*∗*	4.19±0.59

Preoperative diagnosis				
IHD	12(44.45%)	7(38.9%)		19 (42.2%)
Valvular HD	11(40.75%)	6(33.3%)		17 (37.7%)
Cong. HD	3(11.1%)	0	0.074*∗∗∗*	3 (6.6%)
IHD& valvular HD	1(3.7%)	5(27.8%)		6 (13.3%)

*∗*P value was computed by t-test.

*∗∗∗*P value was computed by Chi Square test.

**Table 2:** Comparison of cardiac surgical data between AKI and non-AKI groups.

Parameters	Group 1	Group 2	P value	Total
	n= 27	n= 18		n= 45
Cardiac Procedure n(%)				
CABG	12(44.45%)	10(55.55%)		22 (48.9%)
SVR	6(22.22%)	0	0.074*∗∗∗*	6 (13.2%)
DVR	5(18.5%)	6(33.3%)		11 (23.9%)
ASD	2 (7.4%)	0		2 (4.3%)
SAM	1 (3.7%)	0		1 (2.2%)
CABG + Single Valve S.	1(3.7%)	2 (11.11%)		3 (6.5%)

Duration of CPB (minutes) [Mean±SD]	137.22±31.7	126.16±23.79	0.215*∗*	132.8±29.03

Cross clamping time (minutes) [Median(Min-Max)]	95(60-170)	80(60-120)	0.383*∗∗*	80(60-170)

Core temp (°C) [Median(Min-Max)]	33(32-35)	34(32-35)	0.055*∗∗*	33(32-35)

*∗*P value was computed by t-test.

*∗∗*P value was computed by Mann-Whitney test.

*∗∗∗*P value was computed by Chi Square test.

**Table 3:** Comparison of AKI parameters between the two groups.

	Group 1	Group 2	P value
	n= 27	n= 18	
Pre-operative UOP (ml/12h)	1000(800-1600)	950(800-1100)	0.027*∗∗*

Post-operative UOP (ml/12h)	3696.29±552.77	3416.66±545.83	0.102*∗*

Pre-operative KIM-1 (ng/mg)	1.16±0.76	1.29±0.79	0.602*∗*

Post-operative KIM-1 (ng/mg)	1.70(0.1-3.1)	1.42(0.1-2.1)	0.015*∗∗*

Pre-operative Creatinine(mg/dl)	0.8(0.5-1.4)	0.90(0.7-1.1)	0.868*∗∗*

3h Post-operative creatinine(mg/dl)	0.8(0.6-1.3)	0.8(0.6-1)	0.741*∗∗*

24h Post-operative Creatinine (mg/dl)	1.40(1.1-2.2)	0.95(0.7-1.1)	<0.001*∗∗*

UOP: urine output.

*∗*P value was computed by t-test.

*∗∗*P value was computed by Mann-Whitney test.

*∗∗∗*P value was computed by Chi Square test.

**Table 4:** Performance characteristics of urinary KIM-1 3 h after operation.

	Cut off point	AUC	P-value	Sensitivity	Specificity	PPV	NPV
3h Postoperative KIM-1	1.9	0.715	0.015	48%	94%	92.8%	54.8%

## Data Availability

Data are available once required and will be provided by the corresponding author.
